# Complaining

**Published:** 1871-02

**Authors:** 


### COMPLAINING.

While those who are never well should have our warmest sympathies, very different is it with that innumerable host of chronic growlers who are pouring into our afflicted ears the interminable tale of their sorrows, the whole of which are imaginary, are from mere habit, or are from their own making.

Some persons, after having been sick a short time, are very liable to fall into the habit of complaining, which becomes so inveterate that they unconsciously begin to detail symptoms which long before had ceased to have an existence.

For that unfortunate class who are forever looking out for symptoms, and seem to feel a real gratification to have something to growl about, and will shake their heads into a headache, or poke their sides in search of a "hurt" if they have none, there is no hope; they are clean gone daft. But let the reader take a hint on the subject; except in your own family, no one cares a straw about your complaints, and, for the sake of getting rid of your rigmarole in the shortest time possible, will thrust a piece of advice under your nose which cured somebody a great deal worse than you are, and of course it will cure you; and, having fixed you off, they are then for business.

If you have nothing better than a complaint to say of yourself, hadn't you better go on the principle, "If you havn't anything good to say, say nothing?"

What good can it do for you to be talking about your imperfections? If you are speaking to a real friend, it only saddens him to think that you are suffering, and the more so as he is helpless to aid you. There is some sense in a man's speaking of his actual virtues, or what he has done for the good of others; but to be telling Tom, Dick, and Harry, the instant you lay your eyes on them, that you have a sore toe, or a boil on your body, or the "rheumatiz" in your joints, oi a hole in the spine of your back, man alive, be ashamed of yourself, and from this day forth, if a friend asks you

"HOW D'YE DO?".

say, "Pretty well, I thank you," and then talk about something else. In this way you will at least get rid of trying a thousand and one things which have cured a man "no worse than you," or "a great deal better than you," — we don't know which.

